# Safety and efficacy of Cangrelor versus GPIIb/IIIa inhibitors as adjunctive therapy in endovascular treatment of acute ischemic stroke: a systematic review and meta-analysis

**DOI:** 10.1007/s11239-025-03160-9

**Published:** 2025-08-04

**Authors:** Mohamed Ellebedy, Rashad G. Mohamed, Mina Ihab Lamie, Omar F Abbas, Amir Hegazi, Muataz Kashbour

**Affiliations:** 1 Faculty of Medicine, Sohag University, Sohag, Egypt; 2 Mansoura Manchester Program for Medical Education, Faculty of Medicine, Mansoura University, Mansoura, Egypt; 3 Faculty of medicine, Al-Azhar University, Cairo, Egypt; 4 Diagnostic Radiology Department, National Cancer Institute, Misrata, Libya; 5 Medical Research Group of Egypt (MRGE), Negida Academy LLC, Arlington, MA USA

**Keywords:** Cangrelor, Glycoprotein IIb/IIIa inhibitors, Endovascular treatment, Ischemic stroke, Meta-analysis

## Abstract

Endovascular treatment (EVT) plays a critical role in the management of acute ischemic stroke (AIS). To enhance reperfusion outcomes, intravenous antiplatelet agents such as glycoprotein IIb/IIIa inhibitors (GPIs) are commonly administered during EVT. cangrelor, a fast-acting, reversible P2Y12 inhibitor, has emerged as a potential alternative due to its rapid onset and offset of action. However, comparative data on its efficacy and safety relative to GPIs in this context are limited. This study aimed to systematically evaluate the efficacy and safety of cangrelor versus GPIs as adjunctive antiplatelet therapies during EVT in AIS patients. We conducted a systematic review and meta-analysis following PRISMA guidelines, with protocol registration on PROSPERO (CRD420251047232). A comprehensive literature search was performed in PubMed, Scopus, and Web of Science, through May 4, 2025. Studies comparing cangrelor and GPIs during EVT in AIS were included. Data on procedural success, intracranial hemorrhage, and mortality were extracted and analyzed using Review Manager 5.4. Five studies including 616 participants (cangrelor: 318, GPIs: 298) were included. No significant difference was observed between groups in achieving the modified treatment in cerebral infarction (mTICI) score ≥ 2b (RR = 1.04, 95% CI [1.00–1.09], *P* = 0.07), mTICI ≥ 2c (RR = 1.19 (95% CI [0.87, 1.61], *P* = 0.27), or mTICI ≥ 3 (RR = 1.07 (95% CI [0.81 to 1.42], *p* = 0.62). Safety outcomes, including symptomatic intracranial hemorrhage and mortality, were comparable. Despite limited data, current evidence indicates that cangrelor may serve as a viable alternative to GPIs in the EVT of AIS, with comparable efficacy and safety profiles. However, definitive conclusions require validation through well-designed prospective randomized trials.

**Graphical abstract:**

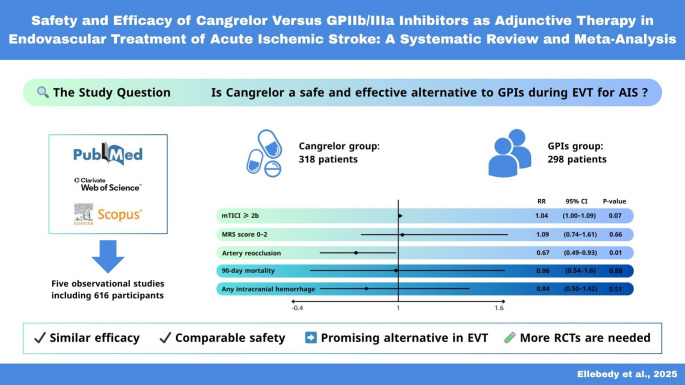

## Introduction

Ischemic stroke, as defined by the American Heart Association/American Stroke Association (AHA/ASA), is an episode of neurological dysfunction resulting from focal cerebral, spinal, or retinal infarction [1]. It remains a significant global health concern, causing over 7.6 million new cases and 3.3 million deaths each year [2]. This emphasizes the importance of adequate and prompt management of patients suffering from acute ischemic stroke (AIS). The AHA/ASA 2019 guidelines for management for AIS include stabilization of the patient’s general condition, intravenous fibrinolysis, and endovascular treatment (EVT) [3]. EVT aims to achieve reperfusion with a modified Thrombolysis in Cerebral Infarction (mTICI) grade 2b or 3 angiographic result to maximize the likelihood of a favorable functional outcome [3, 4]. However, this may be impaired and not achieved in some patients [4, 5] due to different causes that are still not clearly defined but may include new thrombus formation, so the AHA/ASA 2019 guidelines consider the usage of I.V. glycoprotein IIb/IIIa inhibitors (GPIs) during procedure to reduce risk of thrombosis [3].

GPIs work primarily by binding to glycoprotein IIb/IIIa receptors and preventing it from binding either fibrinogen or von Willebrand factor (vWF) which in turn inhibits platelet aggregation and adhesion [6]. Another family of antiplatelet drugs are P2Y12-receptor inhibitors, also called ADP-receptor inhibitors. This class of drugs works by binding to and inhibiting the P2Y12 receptor, a platelet receptor that normally activates a signaling pathway leading to glycoprotein IIb/IIIa receptor activation. Some of these drugs include clopidogrel, prasugrel, ticagrelor, and cangrelor [7].

Cangrelor is a relatively new P2Y12-receptor inhibitor which promises better outcomes and safety as an antiplatelet agent used during endovascular procedures [8]. Thus, further trials compared cangrelor for use during Percutaneous Coronary Intervention (PCI) as an alternative for GPIs with promising results for cangrelor in terms of safety, and with efficacy comparable to that of GPIs [9, 10]. Even more studies investigated the use of cangrelor in EVT of AIS and further supported its safety and efficacy during these procedures [11–14]. However, it remains poorly studied as an alternative for the conventional antiplatelet agents used during endovascular procedures (i.e., GPIs), which may sometimes be limited by cost or availability, during EVT of AIS. So, we aim to shed light on the available data comparing GPIs with cangrelor as a potential alternative.

## Methods

### Protocol registration

Our systematic review and meta-analysis was conducted in accordance with the Preferred Reporting Items for Systematic Review and Meta Analysis (PRISMA) guidelines [15]. Ethical approval and patient consent were not required, as this analysis was based on previously published studies. We registered our research protocol on PROSPERO under the registration number: CRD420251047232.

### Data sources and search strategy

We conducted a systematic search of three electronic databases; PubMed, Scopus, and Web of Science to identify relevant literature published up to May 4, 2025. Our search strategy employed a combination of keywords and medical subject headings combined with Boolean operators “AND” and “OR” to ensure comprehensive coverage of the literature. Specifically, we used the following search strategy: (cangrelor OR kengreal) AND (((gp OR glycoprotein) AND (inhibitor OR antagonist)) OR tirofiban OR aggrastat OR agrastat OR eptifibatide OR epifibatide OR integrilin OR integrelin OR abciximab OR centorx OR reopro OR clotinab)) AND (((endovascular OR (minimally invasive) OR (catheter)) AND (treatment OR therapy OR intervention OR procedure OR revascularization OR thrombectomy OR embolectomy)) OR pci OR (percutaneous coronary intervention)). The search strategy was adjusted to meet the requirements of each database. The results were imported into Rayyan software, a platform designed to facilitate the systematic review process, especially for screening and selecting studies.

### Study selection and eligibility criteria

The inclusion criteria were based on the population, intervention, comparator, and outcome framework. Two independent investigators assessed all articles at the title and abstract level, after which the full text was read to confirm relevance. The conflicts were resolved by discussion. We included studies that compared cangrelor with GPIs as adjunctive therapy in EVT in AIS patients. We excluded studies if they were reviews, animal studies, conference abstracts, or non-English articles. The reference lists of retrieved studies were hand-searched to include additional eligible studies.

### Data extraction

We extracted the following study-level data: first author’s last name, year of publication, study design, data source, study period, country, sample size, cause of stroke, population/inclusion criteria, use of adjuvant therapies or procedures other than antiplatelet therapy (APT), indications for adjunctive intravenous antiplatelet therapy, and the main conclusions. For the intervention group (cangrelor), we collected details including sample size, dosage and route of administration, and timing of administration (pre-, intra-, or post-EVT). For the comparison group, we recorded similar information: sample size, type and dosage of antiplatelet therapy, route and timing of administration.

Baseline characteristics included age, sex distribution, and medical history (hypertension, diabetes, hypercholesterolemia, current smoking, coronary artery disease, prior stroke or transient ischemic attack (TIA), prior antiplatelet or anticoagulant use, pre-stroke mRS > 1), as well as site of occlusion (M1 or M2 segment of the middle cerebral artery).

Efficacy outcomes included rates of mTICI ≥ 2b, mTICI ≥ 2c, mTICI ≥ 3, 90-day mRS 0–2, early neurological improvement, successful reperfusion, and artery re-occlusion at 1 day after procedure. Safety outcomes included in-hospital mortality, 90-day mortality, any intracranial hemorrhage (ICH), symptomatic ICH, and parenchymal hematoma. For all outcomes, we recorded the number of events and the total sample size for each group, as all were dichotomous variables.

To eliminate any overlap between the two studies included in the meta-analysis, we exclusively extracted data from the Milnerowicz er al. 2025 study for patients who had not undergone stenting, as the Pop er al. 2024 study specifically concentrated on stenting cases. This strategy ensured that the patient populations analyzed remained distinct and non-overlapping.

### Risk of bias assessment

Two investigators independently conducted the quality assessment of each included studies, using the Newcastle-Ottawa Scale (NOS), which includes three domains: selection process, comparability assessment, and outcome assessment. Each study’s overall risk of bias was classified as “Poor quality”, “some concerns”, or “Good quality” [16].

### Statistical analysis

Review Manager (RevMan version 5.4) was used for all the statistical analyses with the results pooled using a random effects model, which was chosen to account for heterogeneity between studies. A P-value was considered significant when it was ≤ 0.05. The risk ratios (RRs) were employed to express the dichotomous outcome data, with 95% confidence intervals (CIs). To conduct heterogeneity evaluation, the Higgins I2 statistic value was used, and it was considered significant when it was > 50%.

## Results

### Study selection

**Figure 1:**
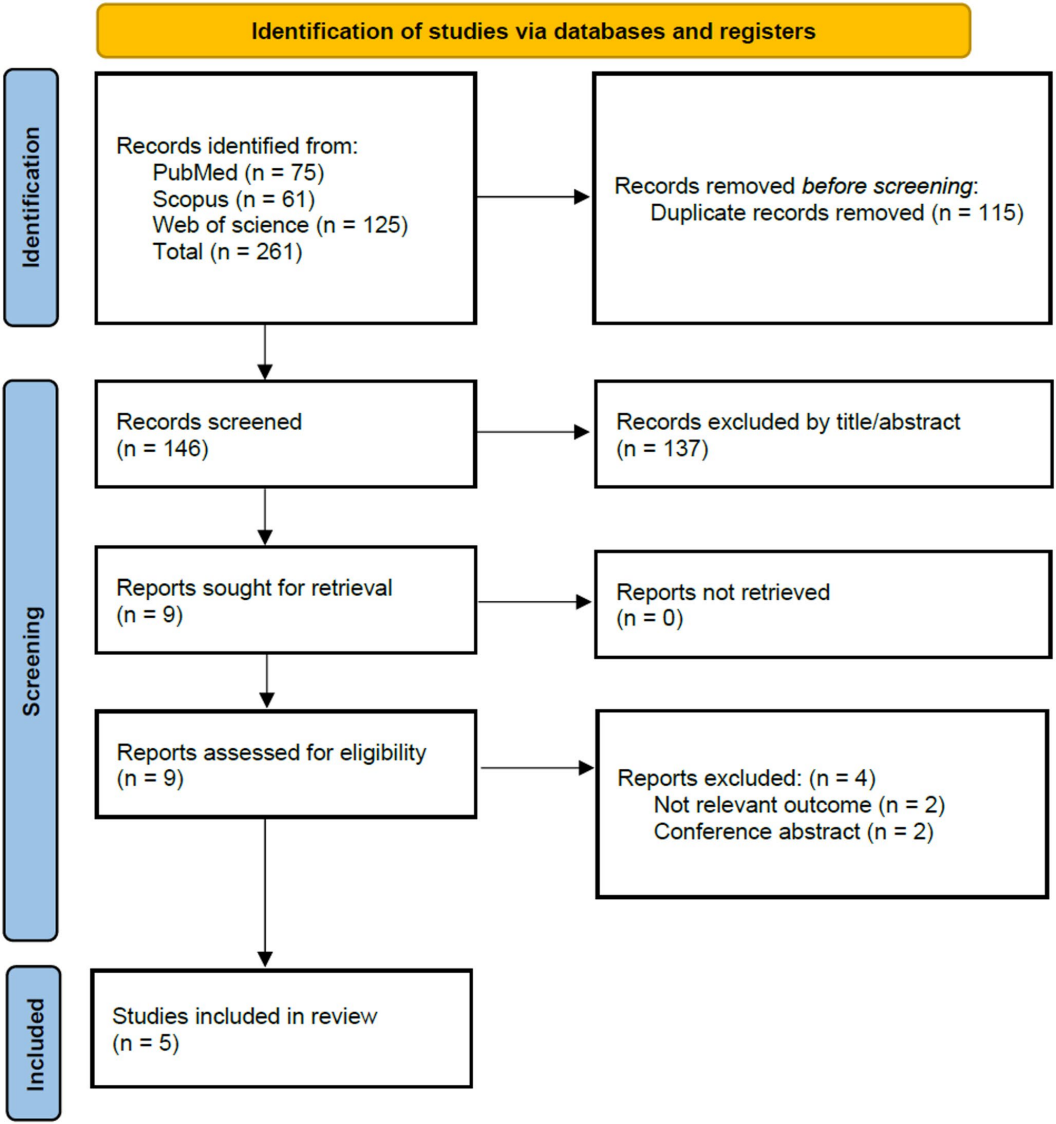
**Fig. 1 **PRISMA flow diagram of study selection

The initial literature search identified 261 records from PubMed (*n* = 75), Scopus (*n* = 61), and Web of Science (*n* = 125). A total of 115 duplicate articles were removed, followed by the exclusion of 137 studies during the title and abstract screening. After a comprehensive full-text screening, we identified 5 studies that met the inclusion criteria for our review (Fig. 1).

### Study characteristics

A total of five retrospective studies, published between 2021 and 2025, were included in this review [17–21]. The studies were conducted across France, the USA, and Spain, with sample sizes ranging from 17 to 559 participants. All studies compared intraoperative or periprocedural use of cangrelor with glycoprotein IIb/IIIa inhibitors during endovascular treatment for acute ischemic stroke. A total of 616 patients were included in the meta-analysis, with 318 assigned to the cangrelor group and 398 to GPIs group. The mean age of participants ranged from 57 to 69 years. Males comprised the majority in both groups, ranging from 60.3 to 88.9%. Median NIHSS scores on admission ranged from 12.5 to 17.5, and pre-treatment DWI-ASPECTS were comparable across studies. Tables 1 and 2 outline the key summary and baseline characteristics of the studies included in our review.

**Table 1:** Summary of the included studies

Study ID	Study design	Data source	Date	Country	Number of participants	Population / inclusion criteria	Adjuvant Therapy or procedures other than APT	Main conclusion	Cangrelor group			Comparison group			
									Sample size	Dosage and administration route	Timing of administration	Sample size	Type	Dosage and administration route	Timing of administration
Milnerowicz et al., 2025	Retrospective	Endovascular Treatment of Ischemic Stroke (ETIS) Registry	Between July 2018 and September 2023	France	559	Acute ischemic stroke patients who underwent EVT with intraoperative cangrelor or GPIIb/IIIa inhibitors, regardless of the indication for antiplatelet therapy	An intravenous bolus of aspirin was administered at the operator’s discretion	Cangrelor showed comparable safety and efficacy to GPIIb/IIIa inhibitors. These results, along with specific pharmacodynamics, make this drug a promising agent in the acute management of complex intracranial and extracranial LVOS	399	It was given only intravenously, starting with a bolus, then a continuous infusion, following standard protocols and local practices.	Intraoperative	160	Tirofiban, abciximab, or eptifibatide	It was given only intravenously, starting with a bolus, then a continuous infusion, following standard protocols and local practices	Intraoperative
Devarajan et al., 2024	Retrospective	A prospectively maintained database	Between December 2018 and March 2023	USA	140	Patients who received cangrelor or eptifibatide intraoperatively while undergoing EVT for management of acute stroke	Placement of an intracranial stent or placement of an extracranial stent	Cangrelor was associated with a decreased risk of hemorrhagic conversion and might lead to favorable functional outcomes for patients during hospitalization in comparison with GPIs	36	Initial bolus of 30 µg/kg was administered Intravenous, followed by a steady infusion rate of 4 µg/kg/min	Intraoperative	104	Eptifibatide	Initial bolus of 0.75 mg/kg or half bolus may be given intra-arterially at the target vessel at the discretion of the provider, followed by a continuous infusion of 1 µg/kg/min	Intraoperative
Pop et al., 2024	Retrospective	Endovascular Treatment in Ischemic Stroke (ETIS) Registry	Between January 2015 and June 2023	France	384	Adults (≥ 18 years) with anterior tandem lesions who underwent thrombectomy with carotid stenting and received intra-procedural antiplatelet therapy (cangrelor, GPIIb/IIIa inhibitor, or aspirin)	Carotid artery stenting & some patients in both groups received a 250–500 mg IV bolus of aspirin, based on center protocol	This study indicates a potential trend towards lower odds of favorable clinical outcomes with cangrelor treatment compared with GPIIb/IIIa inhibitors	91	It was given as an IV bolus followed by infusion, at either full or reduced doses, and infusions were stopped post-procedure or continued up to 24 h based on each center’s protocol.	Intraoperative	77	Tirofiban, eptifibatide, specified	It was given as an IV bolus followed by infusion, at either full or reduced doses, and infusions were stopped post-procedure or continued up to 24 h based on each center’s protocol	Intraoperative
Jumaa et al., 2023	Retrospective	A pooled multicenter cohort registry	NA	USA and Spain	63	Adult patients with tandem lesion treated with mechanical thrombectomy within 24 h after stroke onset	Intraprocedural heparin, ICA artery stenting (for some patients)	Cangrelor has similar safety and an increased rate of complete reperfusion compared to IV GP-IIb/IIIa inhibitors	30	IV 2 mcg/kg/min infusion with 15 mcg/kg bolus	Periprocedural	33	Tirofiban and eptifibatide	Tirofiban: 0.1 mcg/kg/min infusion with no bolus, Eptifibatide: 0.5 to 2 mcg/kg/min infusion with or without 90 mcg/kg bolus	Periprocedural
Delvoye et al., 2021	Retrospective	A prospective collected monocentric registry	Between January 2015 and December 2019	France	17	Acute ischemic stroke patients treated by EVT for an AIS of the anterior circulation	EC-ICA stenting, heparin was administered at the dose of 50UI/kg, if no IV-tPA was given before	When used as a rescue treatment during emergent stenting of ECICA, Cangrelor present a better safety profile than Abciximab, with less intracranial hemorrhages and a higher rate of good clinical outcome	9	An IV bolus of 30 mg/kg followed by a continuous infusion at 4 mg/kg/min and discontinued at the end of the procedure	Perioperative	8	Abciximab	Abciximab: an IV bolus of 0.25 mg/kg followed by a continuous infusion of 0.125 mg/kg/min for 12 h	Perioperative

**Table 2:** Baseline characteristics of the study population

Study ID	Groups	Sample size	Age; years (mean, SD)	Male sex (N, %)	Medical history (N, %)								Pre-stroke mRS > 1 (N, %)	admission NIHSS (median, IQR)	Pre-treament DWI-ASPECTS (median, IQR)	IV thrombolysis (N, %)	Tandem occlusion (N, %)	Site of intracranial occlusion (N, %)			Adjuvant stenting (N, %)
					Hypertension	Diabetes	Hypercholesterolemia	Current smoking	History of coronary artery disease	History of previous stroke or TIA	Prior antiplatelet use	Prior anticoagulant use						M1-MCA	M2-MCA	ICA	
Milnerowicz et al., 2025	Cangrelor	399	65.7 (13.4)	271 (67.9)	227 (57.0)	96 (24.1)	123 (30.7)	121 (30.4)	38 (9.6)	65 (16.3)	102 (25.7)	36 (9.0)	352 (88.2)	13 (7–18)	8 (7–9)	171 (43.0)	126 (31.5)	107 (26.9)	31 (7.8)	62 (15.4)	247 (61.9)
	GPIIb/IIIa inhibitors	160	66.5 (14.4)	102 (63.8)	94 (58.8)	28 (17.7)	46 (28.5)	51 (31.9)	17 (10.9)	21 (13.4)	35 (21.7)	15 (9.4)	145 (90.6)	13 (7–18)	8 (7–9)	41 (25.7)	56 (35.2)	45 (28.1)	12 (7.5)	22 (13.5)	84 (52.8)
Devarajan et al., 2024	Cangrelor	36	65 (15)	24 (66.7)	29 (80.6)	13 (36.1)	15 (41.7)	18 (50)	7 (19.4)	4 (11.1)	10 (27.8)	1 (2.8)	NA	12.5 (9.5–17.0)	9.0 (8.0–10.0)	11 (30.6)	16 (44.4)	NA	NA	NA	34 (94)
	GPIIb/IIIa inhibitors	104	65.1 (13.1)	71 (68.2)	70 (67.3)	41 (39.4)	42 (40.4)	53 (51.0)	19 (18.3)	13 (12.5)	30 (28.8)	4 (3.8)	NA	14.0 (7.0–19.0)	9.0 (8.0–10.0)	27 (26.0)	30 (28.8)	NA	NA	NA	58 (55.8)
Pop et al., 2024	Cangrelor	91	68 (12)	64(60.3)	45 (51.1)	21 (23.3)	29 (32.2)	21 (25.6)	NA	15 (16.7)	24 (26.7)	NA	NA	13 (11)	8 (2)	35 (38.5)	NA	45 (58.4)	14 (18.2)	14 (18.2)	91 (100)
	GPIIb/IIIa inhibitors	77	65 (12)	50(64.9)	36 (50.0)	8 (11.4)	19 (26.8)	28 (38.9)	NA	7 (10.0)	14 (19.4)	NA	NA	13 (9)	8 (2)	23 (30.3)	NA	39 (69.6)	9 (16.1)	8 (14.3)	77 (00)
Jumaa et al., 2023	Cangrelor	30	68.7(7)	21 (70)	23 (76.7)	7 (23.3)	16 (55.2)	9 (30)	5 (16.7)	7 (23.3)	8 (28.6)	NA	4 (13)	17.5 (11–21)	9 (8–10)	NA	30 (100)	17 (94.4)	1 (5.6)	0 (0)	29 (96.7)
	GPIIb/IIIa inhibitors	33	66.3 (10.9)	25 (76)	29 (87.9)	9 (27.3)	20 (60.6)	11 (33.3)	5 (15.2)	6 (18.2)	7 (21.2)	NA	2 (6)	15 (11–18)	8 (6–9)	NA	33 (100)	14 (63.6)	3 (13.6)	5 (22.7)	25 (83.3)
Delvoye et al., 2021	Cangrelor	9	65.3 (19.2)	8 (88.9)	6 (66.7)	3 (33.3)	2 (22.2)	1 (11.1)	NA	NA	3 (33.3)	NA	2 (55.6)	17 (14–21)	7 (6–8)	3 (33.3)	7 (77.8)	4 (44.4)	0 (0)	2 (22.2)	9 (100)
	GPIIb/IIIa inhibitors	8	57.3 (18.8)	7 (87.5)	4 (50.0)	2 (25.0)	4 (50.0)	2 (25.0)	NA	NA	4 (50.0)	NA	2 (25.0)	12 (8–16)	8 (8–8)	4 (50.0)	4 (50.0)	2 (25.0)	1(12.5)	0 (0)	8 (100)

### Risk of bias assessment

We used the Newcastle-Ottawa Scale (NOS) for quality assessment of our five observational studies. Four studies were judged to be of good quality, and only one study was judged to be of poor quality due to a lower rating in the comparability domain. A summary of the risk-of-bias assessment results is presented in Table 3.

**Table 3:** Risk of bias assessment results

study ID	Selection	Comparability	outcome	overall	Final decision
Milnerowicz 2025	4	2	3	9	Good quality
Devarajan 2024	4	2	3	9	Good quality
Pop 2024	4	2	3	9	Good quality
Jumaa 2023	4	2	3	9	Good quality
Delvoye 2021	4	0	3	7	poor quality

### Efficacy outcomes

#### mTICI ≥ 2b

**Figure 2:**
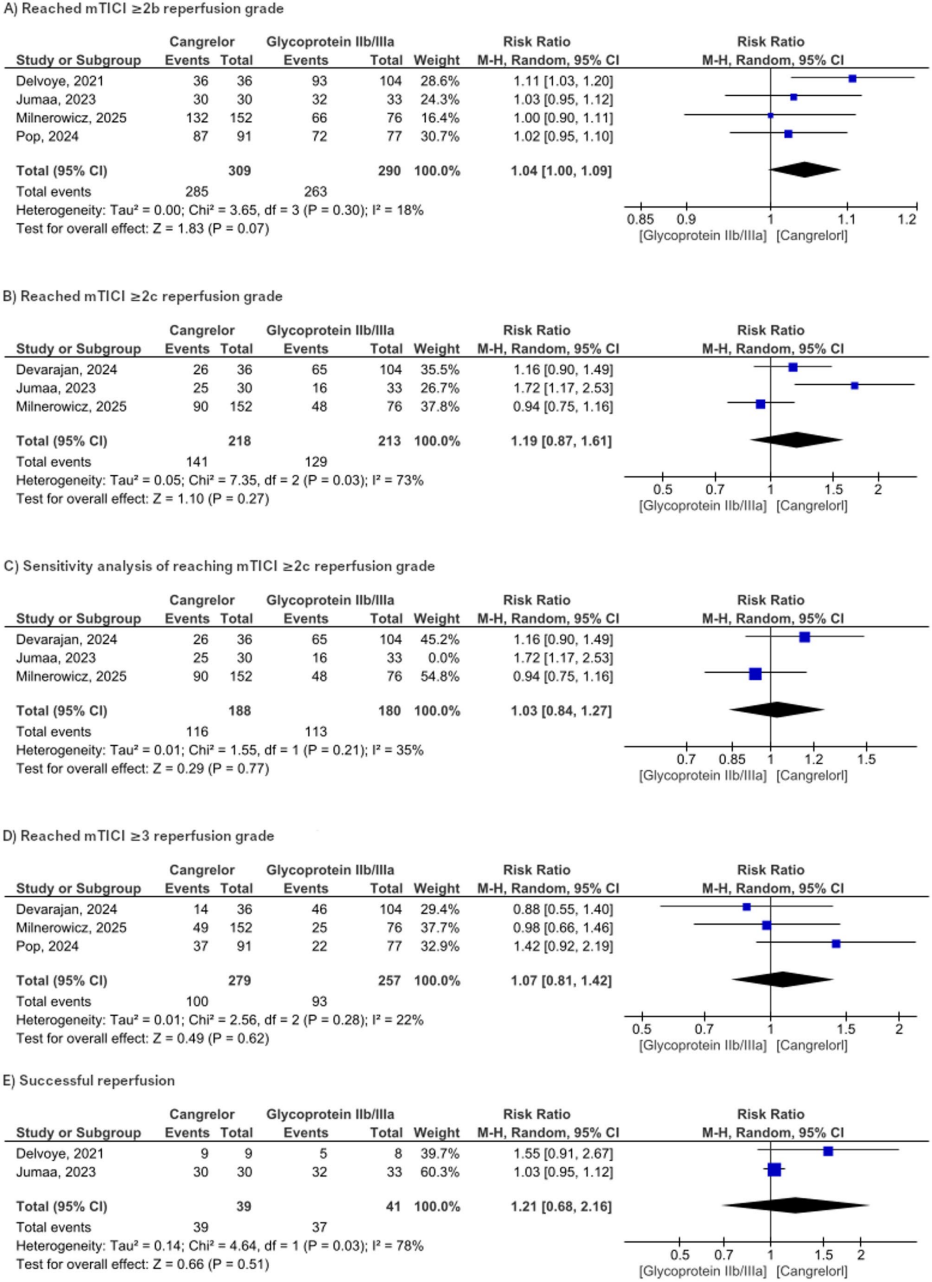
**Fig. 2** Forest plot representation of reperfusion-related results

Four studies evaluated the mTICI ≥ 2b score with a total of 599 participants. The pooled effect did not statistically favor cangrelor over the use of glycoprotein IIb/IIIa inhibitors (RR = 1.04, 95% CI [1.00,1.09], *P* = 0.07, Fig. 2a). No significant heterogeneity was observed (*P* = 0.3, I2 = 18%).

#### mTICI ≥ 2c

The mTICI ≥ 2c was reported in three included studies. Cangrelor failed to show superiority over the glycoprotein IIb/IIIa inhibitors with RR of 1.19 (95% CI [0.87, 1.61], *P* = 0.27, Fig. 2b). There was substantial heterogeneity (*P* = 0.03, I²= 73%). We conducted a sensitivity analysis to explore the source of heterogeneity. The heterogeneity was resolved by excluding Jumaa et al. However, the pooled effect still did not favor the Cangrelor (RR = 1.03 (95% CI [0.84, 1.27], *P* = 0.77, I2 = 35%, Fig. 2c).

#### mTICI ≥ 3

Our analysis included three studies that reported mTICI ≥ 3 scores with 536 participants. The combined RR shows no statistically significant difference between the two groups, the overall RR = 1.07 (95% CI [0.81 to 1.42], *p* = 0.62, Fig. 2d).

#### Successful reperfusion

Stratified analysis of the combination of all reperfusion grades (mTICI 2b, 2c, and 3) included two studies with a combined RR of 1.21 (95% CI [0.68, 2.16], *P* = 0.51), indicating no statistically significant difference between the two groups. The pooled RR was significantly heterogeneous (*P* = 0.03, I2 = 78%, Fig. 2e).

#### mRS score ≤ 2 on day 90

**Figure 3:**
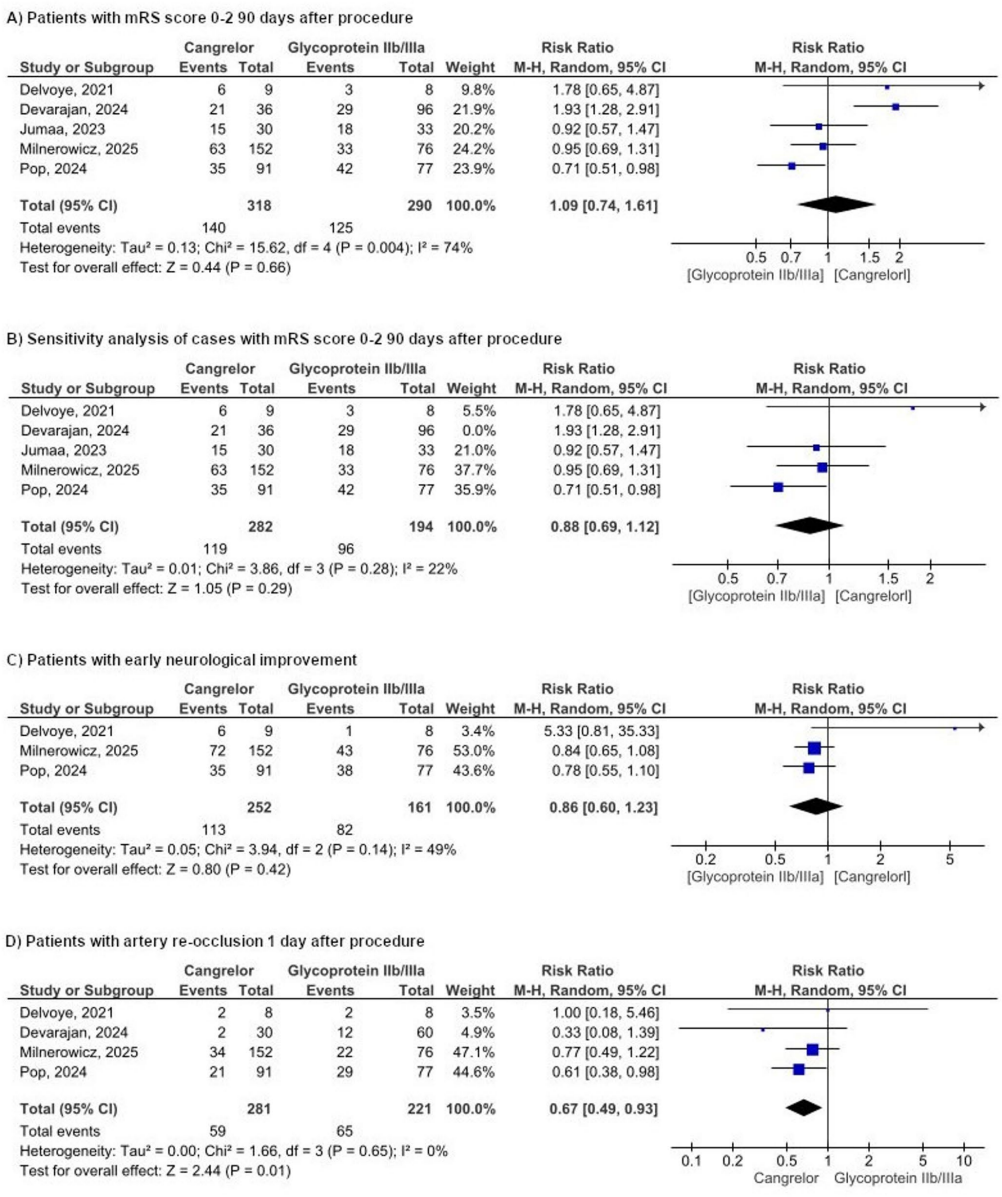
**Fig. 3** Forest plot representation of mortality-related results

Our analysis encompassed five studies that evaluated mRS scores within the range of 0 to 2. The combined RR was 1.09 (95% CI [0.74, 1.61], *p* = 0.66), indicating no statistically significant improvement in the cangrelor group compared to the glycoprotein IIb/IIc inhibitors group (Fig. 3a). The assessment of heterogeneity yielded I2 = 74%, suggesting significant heterogeneity. Sensitivity analysis by excluding Devarajan et al. resolved the heterogeneity (RR = 0.88, 95% CI [0.69, 1.12], *P* = 0.29, I2 = 22%, Fig. 3b).

#### Early neurological improvement (ENI)

Three studies assessed changes in NIHSS scores, either a reduction of 4 or more points in NIHSS or an NIHSS score of 0 to 1 at 24 h. The pooled analysis of the ENI results revealed no significant difference between the cangrelor and the GPIs (RR = 0.86, 95% CI [0.60, 1.23], *P* = 0.42, I² = 49%) (Fig. 3c).

#### Artery re-occlusion at 1 day after procedure

Four studies reported the incidence of re-occlusion after treatment with a total of 502 participants. The pooled RR was significantly lower with Cangrelor than the use of glycoprotein IIb/IIc inhibitors (RR = 0.67, 95% CI [0.49,0.93], *P* = 0.01, Figure). No significant heterogeneity was observed (*P* = 0.65, I2 = 0%) (Fig. 3D).

### Safety outcomes

#### Mortality

**Figure 4:**
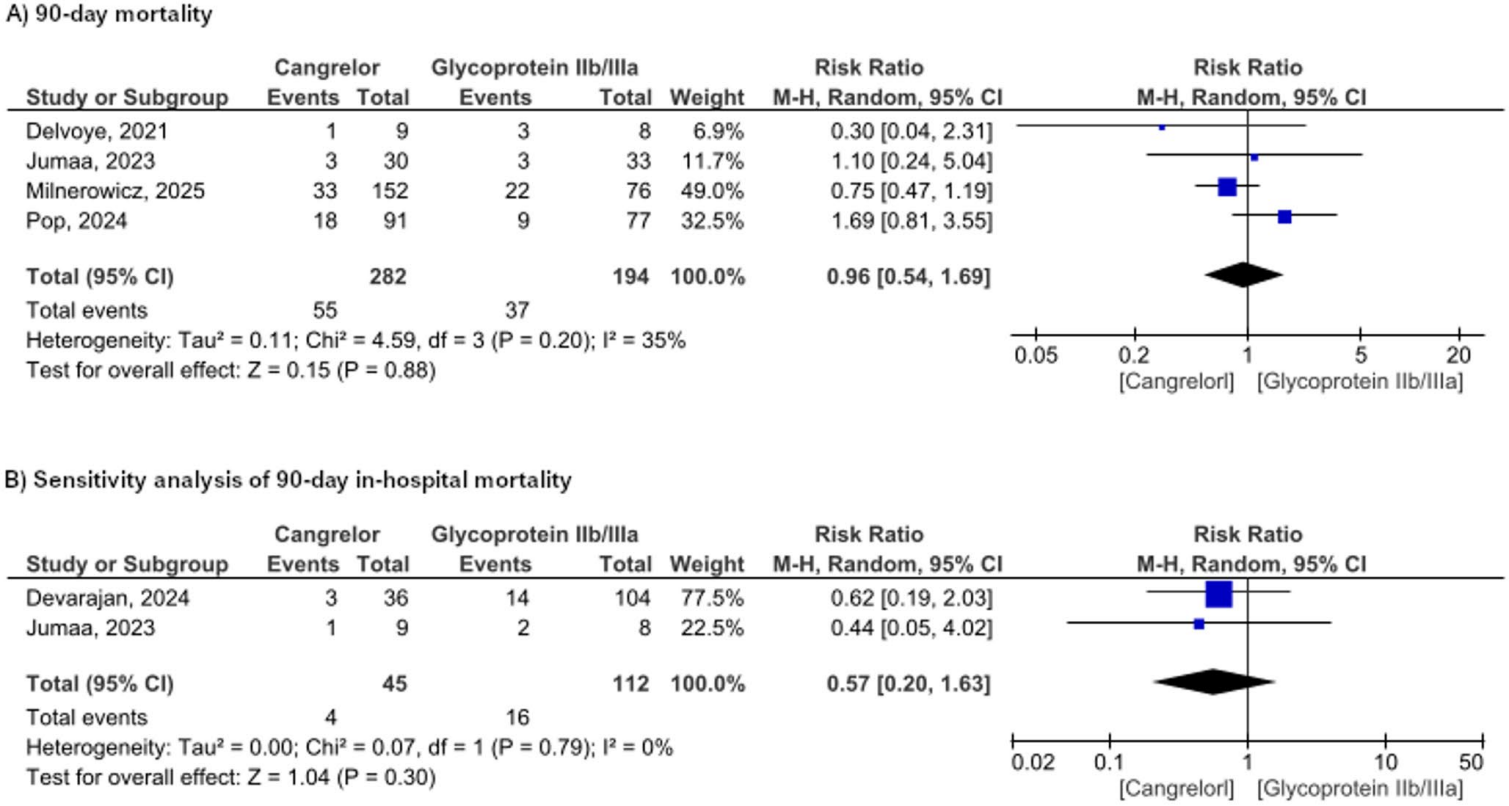
**Fig. 4** Forest plot representation of morbidity-related results

The incidence of mortality at 90 days reported in four studies involving 476 patients showed that cangrelor did not significantly decrease the mortality rate compared to GPIs (RR = 0.96, 95% CI [0.54 to 1.69], *P* = 0.88). There was no heterogeneity among the studies (*p* = 0.20, I² = 35%, Fig. 4a). Furthermore, two studies demonstrate that cangrelor has a lower incidence of in-hospital mortality, although this difference was not statistically significant (RR = 0.57, 95% CI [0.20 to 1.63], *p* = 0.30, Fig. 4b).

#### Any intracranial hemorrhage (ICH)

**Figure 5:**
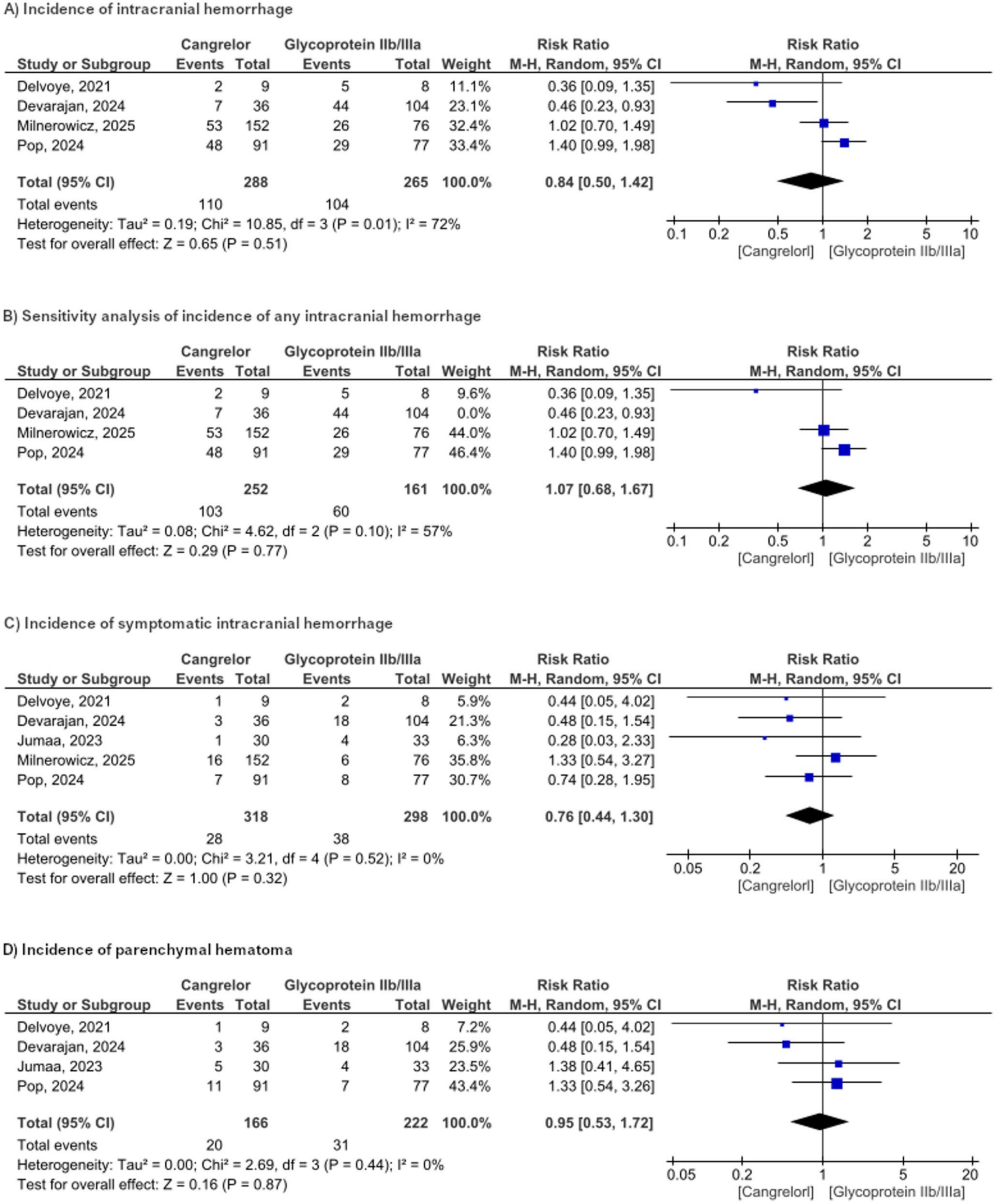
**Fig. 5** Forest plot representation of drug complication-related

The pooled analysis of four studies demonstrated comparable rates of any ICH between the cangrelor and the glycoprotein IIb/IIIa inhibitor groups (RR = 0.84, 95% CI [0.50–1.42], *P* = 0.51, Fig. 5a). Notably, there was substantial heterogeneity (*P* = 0.10, I² = 72%). A sensitivity analysis addressing this heterogeneity, which excluded the study by Devarajan et al. (*P* = 0.1, I2 = 57%) resulted in a reduced but still moderate level of heterogeneity and yielded a new risk ratio of 1.07 (95% CI [0.68–1.67] *P* = 0.77, Fig. 5b).

#### Symptomatic intracranial hemorrhage (sICH)

All studies included in our meta-analysis reported the incidence of sICH. The incidence was lower in the cangrelor group, but this difference was not statistically significant (RR 0.76, 95% CI 0.62–1.28; *p* = 0.53; Figure). No heterogeneity was observed (*P* = 52, I²=0%, Fig. 5c).

#### Parenchymal hematoma (PH)

For PH, the RR did not reach statistical significance different between the cangrelor and GPIs (RR 0.95, 95% CI [0.53,-1.72]; *p* = 0.87; I²=21%, Fig. 5d)

## Discussion

EVT is a cornerstone in the management of complex vascular cases, including acute stroke, intracranial aneurysms, and advanced peripheral arterial disease [22], and the choice of antiplatelet therapy in this context is critical, as it directly influences the balance between preventing vessel re-occlusion (thrombosis) and minimizing bleeding complications [23]. For example, GPIs (e.g., tirofiban and eptifibatide) have been traditionally used to treat thromboembolic complications during endovascular therapy. A potential alternative for GPIs is cangrelor; a unique pharmacologic agent that may have similar or improved safety and efficacy in EVT, particularly for complex or acute neurovascular procedures [24].

To our knowledge, this is the first systematic review and meta-analysis to compare the safety and efficacy of cangrelor versus GPIs as periprocedural antiplatelet agents for EVT in AIS, including data from 616 individuals across five observational studies. Our meta-analysis showed that cangrelor was non-inferior to GPIs in terms of mTICI thresholds (≥ 2b, ≥2c, and ≥ 3), although there was a trend to favor cangrelor in achieving higher degrees of reperfusion, which did not reach statistical significance. However, cangrelor was significantly associated with lower rates of re-occlusion at one day after procedure. Furthermore, both agents demonstrated similar outcomes regarding functional independence at 90 days, early neurological improvement, or mortality, regardless of whether the assessment was made at 90 days or during hospitalization. cangrelor also showed a statistically insignificant effect in reducing the risk of intracranial haemorrhage, symptomatic intracranial haemorrhage, or parenchymal hematoma. However, the absence of increased bleeding risks with cangrelor supports its safety in this setting.

The cause of these results could be the pharmacological profile of the drug. Cangrelor is a reversible, direct-acting P2Y12 receptor antagonist with a rapid onset and short half-life of approximately 3–6 min [24, 25]. This allows for immediate platelet inhibition during the procedure, and after stopping the infusion, the effect on platelets wears off quickly, allowing for precise control during and after procedures, which is advantageous in managing the severity of bleeding events [26]. In contrast, GPIs have longer half-lives and irreversible binding, potentially prolonging bleeding risks post-procedure [27]. Conversely, the similar efficacy in reperfusion outcomes may be due to the comparable potency in preventing thrombus formation during the acute procedural phase, even with different mechanisms. GPIs block fibrinogen binding to the IIb/IIIa receptor [28], while cangrelor inhibits ADP-mediated platelet activation [26]. Therefore, cangrelor may have non-inferior clinical efficacy in achieving complete reperfusion and better functional outcomes compared to GPIs without an increased risk of hemorrhagic complications, suggesting a potential safety advantage [9].

A retrospective, single-centre study from Yerasi et al. 2021 comparing cangrelor and GPIs during PCI showed that cangrelor is safe and effective during PCI, with similar procedural success rates to GPIs but significantly lower major bleeding events and vascular complications [9], which further supports the results of our study. An exploration analysis from the CHAMPION trials by Vaduganathan et al. 2017 demonstrated that cangrelor reduced ischemic events comparably to GPIs and clopidogrel-GPI combinations while showing a trend toward lower severe bleeding and significantly reduced rates of major or minor bleeding [10]. A study by Cervo et al. 2020 on periprocedural cangrelor use in AIS patients requiring stent placement reported that intracranial haemorrhage occurred in 10.5% of patients, with one requiring surgical evacuation and no new hemorrhagic complications within the first week. This study highlights cangrelor’s rapid onset and short half-life as advantages in managing stent-related thrombosis and might be a valuable therapeutic option in the management of emergency cases [11]. A trial registry analysis from Marnat et al. 2022 investigated the safety and efficacy of cangrelor vs. GPIs in acute stroke treated with mechanical thrombectomy and found that cangrelor could be a safe and effective alternative in complex EVTs [12]. Moreover, two different systematic reviews (Desai et al. 2023 and Coulibaly et al. 2024) investigated the safety and efficacy of intravenous dose-adjusted cangrelor for acute neuroendovascular procedures and also showed that intravenous cangrelor appears to be safe and effective in neuroendovascular procedures, with low rates of bleeding and ischemic events [13, 14].

## Limitations

There were several limitations in our study, including heterogeneity among studies, but we attempted to reduce its impact by conducting a sensitivity analysis to exclude studies that had a disproportionate effect on the pooled estimate. Confidence intervals were wide in many outcomes, indicating limited statistical power and the need for larger, more standardized trials. Furthermore, variations existed across studies in stroke severity, occlusion sites, procedural techniques (e.g., stenting, thrombectomy), dosing regimens, and outcome assessment methods. The potential cause of heterogeneity among studies is due to several factors, which include stroke severity and occlusion site variations in baseline characteristics across included studies may influence outcomes. Jumaa et al. 2023 focused on tandem lesions, which may involve more complex or severe occlusions compared to others [19, 29]. We included studies with different endovascular approaches, such as mechanical thrombectomy alone, thrombectomy with stenting, or emergent carotid stenting. The study by Pop et al. 2024 included stenting procedures and may have required prolonged antiplatelet effects [20, 30], therefore potentially favoring GPIs, which reported worse mRS outcomes with cangrelor. The lack of standardized dosing for cangrelor and GPIs across included studies is a significant source of heterogeneity, including differences in bolus versus infusion protocols or timing of administration. The retrospective nature of included studies introduces heterogeneity due to inconsistent reporting and selection biases across studies [31].

Because of the retrospective design of the included studies introducing potential selection bias and variability in treatment protocols and most of the studies being conducted in only three countries, which may affect the generalizability of the findings, we recommend prospective, randomised controlled trials to be conducted to validate these findings, with standardized dosing protocols, consistent outcome definitions, and assessments of long-term functional and safety outcomes. Stratified analyses based on patient characteristics (e.g., stroke severity and occlusion site) and procedural type (e.g., stenting, thrombectomy) are also essential to better define the role of these antiplatelet agents in neurointerventional practice.

## Conclusion

Overall, available data is limited but suggests that cangrelor may be a viable alternative to glycoprotein IIb/IIIa inhibitors as a periprocedural antiplatelet agent during endovascular treatment for acute ischemic stroke, offering comparable benefits in reperfusion success and functional outcomes without increasing hemorrhagic complications. However, more prospective randomized controlled trials are needed to confirm these findings and guide clinical practice.

## Data Availability

No datasets were generated or analysed during the current study.

## Abbreviations

GPIs: Glycoprotein IIb/IIIa inhibitors
AIS: Acute ischemic stroke
EVT: Endovascular treatment
vWF: von Willebrand factor
ICH: Intracranial hemorrhage
sICH: Symptomatic Intracranial Hemorrhage
PH: Parenchymal Hematoma
ENI: Early Neurological Improvement
mTICI: Modified Thrombolysis in Cerebral Infarction score
mRS: Modified Rankin Scale
MCA: Middle cerebral artery
DWI-ASPECTS: Diffusion-Weighted Imaging-Alberta Stroke Program Early Computed Tomography Scores
NIHSS: National Institutes of Health Stroke Scale
RRs: Risk ratios
CIs: Confidence intervals
PRISMA: Preferred Reporting Items for Systematic Review and Meta Analysis
NOS: Newcastle-Ottawa Scale

## References

[1] Sacco RL, Kasner SE, Broderick JP, Caplan LR, Connors JJB, Culebras A et al (2013) An updated definition of stroke for the 21st century: a statement for healthcare professionals from the American heart association/american stroke association. Stroke 44(7):2064–2089 23652265 10.1161/STR.0b013e318296aeca PMC11078537

[2] Feigin VL, Brainin M, Norrving B, Martins S, Sacco RL, Hacke W et al (2022) World stroke organization (WSO): global stroke fact sheet 2022. Int J Stroke 17(1):18–29 34986727 10.1177/17474930211065917

[3] Powers WJ, Rabinstein AA, Ackerson T, Adeoye OM, Bambakidis NC, Becker K et al (2019) Guidelines for the early management of patients with acute ischemic stroke: 2019 update to the 2018 guidelines for the early management of acute ischemic stroke: A guideline for healthcare professionals from the American heart association/american stroke association. Stroke 50(12):e344–418 31662037 10.1161/STR.0000000000000211

[4] Liebeskind DS, Bracard S, Guillemin F, Jahan R, Jovin TG, Majoie CB et al (2019) eTICI reperfusion: defining success in endovascular stroke therapy. J Neurointerv Surg 11(5):433–438 30194109 10.1136/neurintsurg-2018-014127

[5] Goyal M, Menon BK, van Zwam WH, Dippel DWJ, Mitchell PJ, Demchuk AM et al (2016) Endovascular thrombectomy after large-vessel ischaemic stroke: a meta-analysis of individual patient data from five randomised trials. Lancet 387(10029):1723–1731 26898852 10.1016/S0140-6736(16)00163-X

[6] Neumann FJ, Hochholzer W, Pogatsa-Murray G, Schömig A, Gawaz M (2001) Antiplatelet effects of abciximab, Tirofiban and Eptifibatide in patients undergoing coronary stenting. J Am Coll Cardiol 37(5):1323–1328 11300442 10.1016/s0735-1097(01)01165-2

[7] Wallentin L (2009) P2Y(12) inhibitors: differences in properties and mechanisms of action and potential consequences for clinical use. Eur Heart J 30(16):1964–1977 19633016 10.1093/eurheartj/ehp296

[8] Abtan J, Steg PG, Stone GW, Mahaffey KW, Gibson CM, Hamm CW et al (2016) Efficacy and safety of Cangrelor in preventing periprocedural complications in patients with stable angina and acute coronary syndromes undergoing percutaneous coronary intervention: the CHAMPION PHOENIX trial. JACC Cardiovasc Interv 9(18):1905–1913 27659566 10.1016/j.jcin.2016.06.046

[9] Yerasi C, Case BC, Chezar-Azerrad C, Forrestal BJ, Medranda GA, Shea C et al (2021) Cangrelor vs. glycoprotein iib/iiia inhibitors during percutaneous coronary intervention. Am Heart J 238:59–65 33961829 10.1016/j.ahj.2021.04.013

[10] Vaduganathan M, Harrington RA, Stone GW, Deliargyris EN, Steg PG, Gibson CM et al (2017) Evaluation of ischemic and bleeding risks associated with 2 parenteral antiplatelet strategies comparing Cangrelor with glycoprotein iib/iiia inhibitors: an exploratory analysis from the CHAMPION trials. JAMA Cardiol 2(2):127–135 27902833 10.1001/jamacardio.2016.4556

[11] Cervo A, Ferrari F, Barchetti G, Quilici L, Piano M, Boccardi E et al (2020) Use of Cangrelor in cervical and intracranial stenting for the treatment of acute ischemic stroke: A real life Single-Center experience. AJNR Am J Neuroradiol 41(11):2094–2099 33033047 10.3174/ajnr.A6785 PMC7658814

[12] Marnat G, Finistis S, Delvoye F, Sibon I, Desilles JP, Mazighi M et al (2022) Safety and efficacy of Cangrelor in acute stroke treated with mechanical thrombectomy: endovascular treatment of ischemic stroke registry and Meta-analysis. AJNR Am J Neuroradiol 43(3):410–415 35241418 10.3174/ajnr.A7430 PMC8910798

[13] Coulibaly NJ, Elgouhari MH, Arshad MH, Waqas M, Shallwani H, Shakir HJ (2024) Cangrelor for neurointerventional procedures: A systematic review. Interv Neuroradiol.;15910199241247256 10.1177/15910199241247255 PMC11571310 38613377

[14] Desai H, Al-Salihi MM, Morsi RZ, Vayani OR, Kothari SA, Thind S et al (2023) Intravenous Cangrelor use for neuroendovascular procedures: a two-center experience and updated systematic review. Front Neurol 14:1304599 38116108 10.3389/fneur.2023.1304599 PMC10728671

[15] Page MJ, McKenzie JE, Bossuyt PM, Boutron I, Hoffmann TC, Mulrow CD et al (2021) The PRISMA 2020 statement: an updated guideline for reporting systematic reviews. BMJ.;n71 10.1136/bmj.n71 PMC8005924 33782057

[16] Lo CK-L, Mertz D, Loeb M (2014) Newcastle-Ottawa scale: comparing reviewers’ to authors’ assessments. BMC Med Res Methodol 14:45 24690082 10.1186/1471-2288-14-45 PMC4021422

[17] Delvoye F, Maier B, Escalard S, Labreuche J, Thion L-A, Aknouche S et al (2021) Antiplatelet therapy during emergent extracranial internal carotid artery stenting: comparison of three intravenous antiplatelet perioperative strategies. J Stroke Cerebrovasc Dis 30(2):105521 33310073 10.1016/j.jstrokecerebrovasdis.2020.105521

[18] Milnerowicz M, Desilles J-P, Pop R, Dargazanli C, Labreuche J, Sibon I et al (2025) Cangrelor versus gpiib/iiia inhibitors as adjunctive therapy in endovascular treatment of large vessel occlusion stroke. J Neurointerv Surg 10.1136/jnis-2025-023260 40316319

[19] Jumaa MA, Rodriguez-Calienes A, Dawod G, Vivanco-Suarez J, Hassan AE, Divani AA et al (2023) Low dose intravenous Cangrelor versus glycoprotein iib/iiia inhibitors in endovascular treatment of tandem lesions. J Stroke Cerebrovasc Dis 32(12):107438 37883826 10.1016/j.jstrokecerebrovasdis.2023.107438 PMC11271813

[20] Pop R, Finitsis SN, Marnat G, Derraz I, Cognard C, Calviere L et al (2025) Cangrelor for emergent carotid stenting during stroke thrombectomy: a comparative analysis versus glycoprotein iib/iiia inhibitors or aspirin monotherapy. J Neurointerv Surg 10.1136/jnis-2024-022125 39242196

[21] Devarajan A, Gottiparthi S, Caton MT, Ouf A, Wu K, Goldman D et al (2025) Safety and efficacy of Cangrelor in endovascular thrombectomy compared with glycoprotein iib/iiia inhibitors. J Neurointerv Surg 10.1136/jnis-2024-022228 39481883

[22] Mokin M, Jovin TG, Sheth SA, Nguyen TN, Asif KS, Hassan AE et al (2025) Endovascular therapy in patients with acute ischemic stroke with large infarct: A guideline from the society of vascular and interventional neurology. SVIN.;5(2) 10.1161/SVIN.124.001581 PMC12671639 41573174

[23] an de Graaf RA, Zinkstok SM, Chalos V, Goldhoorn R-JB, Majoie CB, van Oostenbrugge RJ et al (2021) Prior antiplatelet therapy in patients undergoing endovascular treatment for acute ischemic stroke: results from the MR CLEAN registry. Int J Stroke 16(4):476–485 32791940 10.1177/1747493020946975 PMC8193619

[24] De Luca L, Steg PG, Bhatt DL, Capodanno D, Angiolillo DJ (2021) Cangrelor: clinical data, contemporary use, and future perspectives. J Am Heart Assoc 10(13):e022125 34212768 10.1161/JAHA.121.022125 PMC8403274

[25] Marcano AL, Ferreiro JL (2016) Role of new antiplatelet drugs on cardiovascular disease: update on Cangrelor. Curr Atheroscler Rep 18(11):66 27714642 10.1007/s11883-016-0617-y

[26] Prüller F, Bis L, Milke OL, Fruhwald F, Pätzold S, Altmanninger-Sock S et al (2018) Cangrelor induces more potent platelet Inhibition without increasing bleeding in resuscitated patients. J Clin Med.;7(11) 10.3390/jcm7110442 PMC6262477 30445678

[27] Hindricks G, Potpara T, Dagres N, Arbelo E, Bax JJ, Blomström-Lundqvist C et al (2021) 2020 ESC guidelines for the diagnosis and management of atrial fibrillation developed in collaboration with the European association for Cardio-Thoracic surgery (EACTS): the task force for the diagnosis and management of atrial fibrillation of the European society of cardiology (ESC) developed with the special contribution of the European heart rhythm association (EHRA) of the ESC. Eur Heart J 42(5):373–498 32860505 10.1093/eurheartj/ehaa612

[28] Vorchheimer DA, Badimon JJ, Fuster V (1999) Platelet glycoprotein iib/iiia receptor antagonists in cardiovascular disease. JAMA 281(15):1407–1414 10217057 10.1001/jama.281.15.1407

[29] Poppe AY, Jacquin G, Roy D, Stapf C, Derex L (2020) Tandem carotid lesions in acute ischemic stroke: mechanisms, therapeutic challenges, and future directions. AJNR Am J Neuroradiol 41(7):1142–1148 32499251 10.3174/ajnr.A6582 PMC7357657

[30] Angiolillo DJ, Galli M, Collet J-P, Kastrati A, O’Donoghue ML (2022) Antiplatelet therapy after percutaneous coronary intervention. EuroIntervention 17(17):e1371–e1396 35354550 10.4244/EIJ-D-21-00904 PMC9896394

[31] Page MJ, McKenzie JE, Kirkham J, Dwan K, Kramer S, Green S et al (2014) Bias due to selective inclusion and reporting of outcomes and analyses in systematic reviews of randomised trials of healthcare interventions. Cochrane Database Syst Rev 2014(10):MR000035 25271098 10.1002/14651858.MR000035.pub2 PMC8191366

